# LncRNA HOX transcript antisense RNA mitigates cardiac function injury in chronic heart failure via regulating microRNA‐30a‐5p to target KDM3A

**DOI:** 10.1111/jcmm.17160

**Published:** 2022-01-26

**Authors:** Xiaoyun Zhang, Yakun Gao, Hongyu Wu, Yong Mao, Yanqing Qi

**Affiliations:** ^1^ Cardio‐Vascular Surgery Ningbo First Hospital Ningbo Zhejiang China

**Keywords:** Bcl‐2/adenovirus E1B 19kDa interacting protein 3, cardiac function, chronic heart failure, long noncoding RNA HOX transcript antisense RNA, lysine‐specific demethylase 3A, microRNA‐30a‐5p

## Abstract

Long noncoding RNA HOX transcript antisense RNA (HOTAIR) has been studied in multiple diseases, but the role of HOTAIR on chronic heart failure (CHF) through the regulation of microRNA (miR)‐30a‐5p and lysine‐specific demethylase 3A (KDM3A) remains unexplored. This research aims to probe the effects of HOTAIR on CHF progression via modulating miR‐30a‐5p to target KDM3A. CHF mouse model was established by intraperitoneal injection of doxorubicin. The CHF mice were then injected with high‐expressed HOTAIR, miR‐30a‐5p or KDM3A adenovirus vectors to determine the cardiac function, oxidative stress, inflammatory response, pathological change and cardiomyocyte apoptosis. HOTAIR, miR‐30a‐5p, KDM3A and Bcl‐2/adenovirus E1B 19kDa interacting protein 3 (BNIP3) expression in CHF mice was detected. The binding relations among HOTAIR, miR‐30a‐5p and KDM3A were validated. HOTAIR and KDM3A were depleted, while miR‐30a‐5p was augmented in CHF mice. The elevated HOTAIR or KDM3A or could improve cardiac function, mitigate oxidative stress and pathological change, reduce inflammatory factor levels and cardiomyocyte apoptosis, while the increased miR‐30a‐5p exerted opposite effects. The miR‐30a‐5p elevation could reverse the effects of enriched HOTAIR, while BNIP3 reduction abrogated the effects of KDM3A on CHF. HOTAIR sponged miR‐30a‐5p that targeted KDM3A. HOTAIR improves cardiac injury in CHF via modulating miR‐30a‐5p to target KDM3A. This study provides novel therapeutic strategies for CHF treatment.

## 1 INTRODUCTION

Heart failure (HF) is a chronic disease that is featured by the incapability of the heart to offer the peripheral tissues with the necessitated amount of metabolism required blood and oxygen.^1^ The occurrence and prevalence of HF are closely associated with age and HF usually afflicts people with coronary artery disease.^2^ Furthermore, high blood pressure, diabetes, obesity, smoking and genetic factors are also accounted for HF incidence; most HF patients suffer from weakness or exhaustion, trouble breathing, weight gaining, leg or abdominal swelling.^3^ There are many newly developed drugs for HF treatment, for instance, diuretics, inotropic agents, neurohumoural inhibitors, vasodilators and ionic channel modulators.^4^ Even though, chronic heart failure (CHF) still remains a major inducer for high mortality and poor quality of life as well as poses staggering economic burden on society. Therefore, it is necessary to further explore novel effective therapeutic strategies for HF.

Long noncoding RNAs (LncRNAs) act as indispensable modulators for multiple biological functions at epigenetic, transcriptional and post‐transcriptional processes; furthermore, lncRNAs are also correlated to the progression of cardiovascular diseases like HF.^5^ As a newly discovered lncRNA, HOX transcript antisense RNA (HOTAIR) plays a pivotal role in gene modulation and the progression of various cancers and diseases such as cardiovascular diseases.^6^ In end‐ and non‐end‐stage HF patients, HOTAIR has been confirmed to be modulated in a concordant manner. In addition, in cardiac hypertrophy that induces HF, HOTAIR is capable of repressing hypertrophy in cultured cardiomyocytes treated with angiotensin II (Ang II). HOTAIR was also predicated to target microRNA (miR)‐30a‐5p on the bioinformatics website. MicroRNAs (miRNAs) are increasingly emerging as crucial involvers for multiple pathophysiological processes that related to HF, such as cardiac fibrosis and hypertrophy. MiR‐30a‐5p exhibits the potential diagnostic value as the biomarker for HF. The failing hearts always absorb miR‐30a‐5p, which is associated with the pathophysiology of HF through targeting related pathways. Moreover, miR‐30a also displays high diagnostic accuracy for HF. In addition, it was predicted through a bioinformatics website that miR‐30a‐5p possessed a target gene lysine‐specific demethylase 3A (KDM3A). KDM3A is an important histone demethylase that regulates gene expression. KDM3A also has been regarded as a potentially safe and effective therapeutic target to prevent the incidence of HF.^7^ Furthermore, it has been speculated that KDM3A may mediate Bcl‐2/adenovirus E1B 19 kDa interacting protein 3 (BNIP3) expression through modulating the methylation of histones on the BNIP3 promoter region. Nevertheless, the studies for probing the regulatory mechanism of HOTAIR, miR30a‐5p and KDM3A on CHF remain inadequate. Therefore, this study further explored the impacts of HOTAIR on the cardiac dysfunction in CHF mice via regulating miR‐30a‐5p and KDM3A, affording novel therapeutic candidates for the treatment of CHF.

## 2 MATERIAL AND METHODS

### 2.1 Ethics statement

Animal experiments were conformed to the Guide to the Management and Use of Laboratory Animals issued by the National Institutes of Health. The protocol of animal experiments was approved by the Institutional Animal Care and Use Committee of Ningbo First Hospital.

### 2.2 Animal model establishment

C57BL/6J male mice aged 6–8 weeks were purchased from the experimental animal centre of Zhejiang Univerisy (Zhejiang, China) for the experiment. The mice were freely provided with water and food in a clean animal laboratory at 18–22°C, with a day and night light cycle. At the 1st week, mice were treated with adaptive feeding.

Doxorubicin (10 mg, DOX; Pu De Pharma) was dissolved in 5 ml normal saline to obtain 2 mg/ml pharmaceutical solution. CHF mice were injected intraperitoneally once a week at a concentration of 5 mg/kg for six consecutive weeks (at the 2nd–7th week).^8^ The Sham group was given an equal amount of saline once a week with total six injections. The CHF mouse modelling was validated by echocardiography and detection of plasma brain natriuretic peptide (BNP, an HF marker). When left ventricular end‐diastolic pressure (LVEDP) ≥15 mmHg and BNP concentration ≥90 pg/ml, the CHF mouse model was considered to be successfully constructed.

### 2.3 Animal treatment

After successful modelling (at the 8th week), CHF mice were anaesthetized, then the pericardium of the mice was removed by left anterior thoracotomy. Thereafter, 100 μl adenovirus solution (with a concentration of 2 × 10^8^ plaque‐forming unit) was injected into the left ventricular of the mice using a microsyringe. The animals were classified into the following groups: the Sham group, the CHF group, the overexpression (oe)‐negative control (NC) group (injected with high‐expressed HOTAIR NC vectors), the oe‐HOTAIR group (injected with high‐expressed HOTAIR vectors), the agomir‐NC group (injected with miR‐30a‐5p high‐expressed NC vectors), the agomir‐miR‐30a‐5p group (injected with high‐expressed miR‐30a‐5p vectors), the oe‐NC +agomir‐NC group (injected with high‐expressed HOTAIR NC and miR‐30a‐5p high‐expressed NC vectors), the oe‐HOTAIR +agomir‐NC group (injected with high‐expressed HOTAIR and miR‐30a‐5p high‐expressed NC vectors), the oe‐HOTAIR +agomir‐miR‐30a‐5p group (injected with high‐expressed HOTAIR and high‐expressed miR‐30a‐5p vectors), the oe‐NC +sh‐NC group (injected with high‐expressed KDM3A NC and low‐expressed BNIP3 NC vectors), the oe‐KDM3A + sh‐NC group (injected with high‐expressed KDM3A and low‐expressed BNIP3 NC vectors) and the oe‐KDM3A + sh‐BNIP3 (injected with high‐expressed KDM3A and BNIP3 vectors). There were 12 mice in each group. All adenovirus vectors used in the experiment were designed and synthesized by GenePharma Co. Ltd.

The follow‐up experiments were performed in mice at the 12th week.

### 2.4 Cardiac function assessment

The electrocardiogram was obtained using the Vevo770 system (VisualSonics) in mice to evaluate cardiac function. Mice were anaesthetized by intraperitoneal injection of 50 mg/kg sodium pentobarbital and their chest hair was removed. Then, the mice were lain on their backs on a special table, and the outcomes of M‐mode tracking were recorded at the baseline. The left ventricular internal diastolic diameter (LVIDd), left ventricular internal systolic diameter (LVIDs), ejection fraction (EF%) and fraction shortening (FS) were measured in mice.

Additionally, the carotid artery of mice was inserted with a 1.4F conductance micromanometer catheter (Millar Instruments) after anaesthesia. When the catheter was inserted into the left ventricle, the PowerLab data acquisition system (AD Instruments) was used to examine the left ventricular maximum/maximum rate of the arterial pressure increase during systole [LV + dP/dt_max_]) and the left ventricular maximum rate of the arterial pressure decrease during systole [LV−dP/dt_max_]) as well as the LVEDP.

### 2.5 Detection of cardiac function‐related factors

The abdominal aortic blood of mice was centrifuged at 1358 × *g* to obtain the supernatant for detection. Cardiac function‐related factors were examined by the enzyme‐linked immunosorbent assay (ELISA) kit from Elabscience Biotechnology. The BNP, cardiac troponin I (CTn‐I) and Ang II contents were detected.

### 2.6 Evaluation of oxidative stress

The mice were anaesthetized with 50 mg/kg pentobarbital sodium and euthanized to obtain hearts. Heart tissues were homogenized and centrifuged at 10,000 × *g* for 20 min at 4°C, The supernatants were harvested and stored at ‐20°C. The activity of superoxide dismutase (SOD) and the content of malondialdehyde (MDA) were measured by the ELISA kit from MSKBIO. The absorbance of the sample was determined by a microplate reader (Millipore).

### 2.7 Inflammatory factor detection

Inflammation factor levels in the blood of mice were evaluated using an ELISA kit purchased from Invitrogen. First, the abdominal aortic blood of the mice was obtained and centrifuged at 1358 × *g* and the precipitate was discarded. Then, the contents of tumour necrosis factor‐α (TNF‐α), interleukin (IL)‐1β and IL‐6 in the supernatant were detected by the ELISA kit.

### 2.8 Histopathological observation

Mice were euthanized after anesthesia to cut off their hearts, which were then placed in 4% paraformaldehyde to be fixed for 24 h. Thereafter, the specimens were embedded in paraffin, cut into 5 μm sections, stained with hematoxylin for 5 min and with eosin for 5 min. After staining, the sections were decolorized rapidly in 95% ethanol, permeabilized with xylene, sealed with the neutral resin and observed under a light microscope to assess the extent of cardiomyocyte damages.

The mouse myocardium was also treated with Masson trichrome staining. Myocardium was fixed in 4% paraformaldehyde, embedded in paraffin and dehydrated, then cut into 5 μm sections and treated with Masson trichrome staining. The outcomes were observed using a light microscope, and the collagen volume fraction (CVF) was calculated by ImageJ software (National Institutes of Health). CVF = collagen area/total viewing area ×100%.

### 2.9 Transferase‐mediated deoxyuridine triphosphate‐biotin nick end labelling (TUNEL) staining

The myocardium apoptosis were determined by terminal deoxynucleotidyl transferase dUTP nick end labeling (TUNEL) assay, using the TUNEL assay kit (R&D, Switzerland). Nuclei were counter‐stained with 4,6‐diamidino‐2‐phenylindole (DAPI). Apoptotic cells were counted in five random fields under a fluorescence microscope. The percent of TUNEL‐positive cells in total myocardial cells was calculated.^9^

### 2.10 Chromatin immunoprecipitation assay

HL‐1 mouse cardiomyocytes were subjected to the chromatin immunoprecipitation (ChIP) assay. The cells were fixed with 1% formaldehyde for 10 min to cross‐link the DNA and protein in the cells. After cross‐linking, the cells were randomly ruptured by ultrasound treatment. Then, the cells were centrifuged at 6540 *g* at 4°C to obtain the supernatant. The positive control antibody RNA polymerase II, NC antibody Immunoglobulin G (IgG) (Abcam) and KDM3A antibody (Novus Biologicals) were added to immunoprecipitate DNA/protein complexes. After immunoprecipitation, DNA was washed and reversely crosslinked; protein was removed using protease K. Finally, BNIP3 promoter level was verified by ChIP kit (Millipore) and reverse transcription quantitative polymerase chain reaction (RT‐qPCR).^10^

### 2.11 Dual luciferase reporter gene assay

HOTAIR wild‐type (WT), HOTAIR mutant (MUT), KDM3A‐WT and KDM3A‐MUT pmiRGLO luciferase expression vector (GenePharma Co. Ltd.) that contained miR‐30a‐5p binding site were used in experiments. These vectors were transfected into HL‐1 cells that had transfected with mimic‐NC or mimic‐miR‐30a‐5p by Lipofectamine 2000 reagent (Invitrogen). The cells were lysed 48 h after transfection, and then the activity of luciferase was examined by a dual luciferase assay system (Promega).^11^

### 2.12 RNA immunoprecipitation assay

The experiment was performed using the RNA immunoprecipitation (RIP)‐binding protein immunoprecipitation kit (Millipore). Cardiomyocytes were lysed in RNA lysis buffer that contained protease and RNase inhibitors. Cell extracts were then incubated with the RIP immunoprecipitation buffer containing magnetic beads conjugated with Ago2 antibody (1:30; Abcam) or IgG antibody (Abcam). After incubation, the samples were incubated with protease K to eliminate residual unbound proteins and isolate immunoprecipitated RNA. RT‐qPCR was performed to detect the levels of HOTAIR, miR‐30a‐5p and KDM3A in the precipitate.

### 2.13 RNA pull‐down assay

The biotinylated miR‐30a‐5p WT, miR‐30a‐5p‐MUT and negative control Bio‐NC were transfected into cells, respectively. Forty‐eight hours later, the cell lysates were incubated with M‐280 streptomyces magnetic beads. Thereafter, HOTAIR expression in the RNA complex that combined with beads was assessed.^12^

### 2.14 RT‐qPCR

The total RNA was extracted using a Trizol kit (Invitrogen). RNA was reversely transcribed into complementary DNA (cDNA) using Mir‐X miRNA RT‐qPCR TB Green® Kit (TaKaRa) or PrimeScript^TM^ RT Master Mix (TaKaRa). The PCR assay was performed by ABI Prism^®^7300 real‐time fluorescence quantitative PCR instrument (Applied Biosystems). U6 and glyceraldehyde‐3‐phosphate dehydrogenase (GAPDH) were set as endogenous controls. The relative levels were calculated by the 2^−∆∆Ct^ method. The relevant primer sequences are listed in Table S1.

### 2.15 Western blot assay

The protein was extracted using RIPA lysis buffer (Sangon Biotech) added with protease inhibitor. The bicinchoninic acid protein determination method was used to detect the protein concentration. Then, 25 μg protein sample was taken, treated with sodium dodecyl sulphate polyacrylamide gel electrophoresis and transferred to polyvinylidene fluoride membrane in transfer buffer. The membrane was sealed with 5% skim milk for 1 h, and then incubated overnight at 4°C with the following primary antibodies: KDM3A (1:2000; Novus Biologicals), BNIP3 (1:1000; Abcam) and GAPDH (1:500; Abcam). Thereafter, the membrane was incubated with horseradish peroxidase‐labelled secondary antibody IgG (1:1000; Abcam) for 2 h. The protein blot visualization was achieved through an ECL Plus Western blot analysis system. GAPDH was set as the internal control.^13^

### 2.16 Statistical analysis

The SPSS 22.0 software (IBM) was used for data analysis. The results were expressed as mean ±standard deviation. The unpaired t‐test was used for the comparison between two groups; one‐way analysis of variance (ANOVA) was used for comparisons among multiple groups and Tukey's post hoc test was used for pairwise comparisons after one‐way ANOVA. *P* < 0.05 was an indicator of statistical significance.

## 3 RESULTS

### 3.1 CHF mice exhibit impaired cardiac function and increased inflammatory respons**e**

**FIGURE 1:**
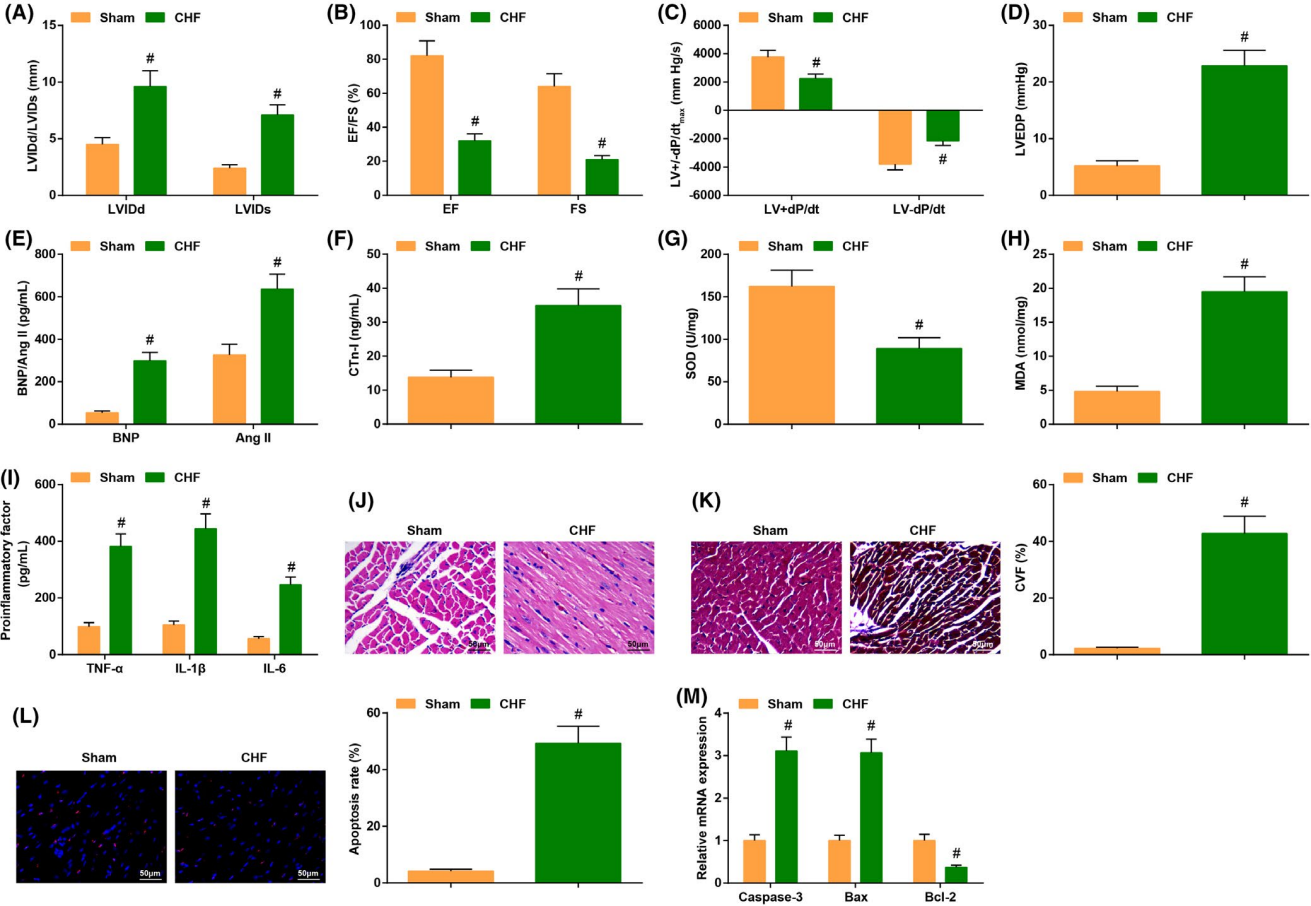
CHF mice exhibit impaired cardiac function and increased inflammatory response. (A–D) The cardiac function in CHF mice was detected by echocardiography; (E–I) the contents of cardiac function‐related factors and inflammatory factors as well as the oxidative stress response were examined by ELISA; (J) the cardiomyocyte damage was examined by HE staining; (K) cardiomyocyte fibrosis was detected by Masson trichrome staining; (L) the apoptosis rate was examined by TUNEL staining; (M) the levels of apoptosis‐related genes were assessed by RT‐qPCR. The data in the figure were all measurement data, and the value was expressed as mean ± standard deviation; the unpaired *t*‐test was used for the comparison between two groups (Fig. G–H, J–M: *n* = 6; remaining figures: *n* = 12); # *p* < .05 vs. the Sham group

We previously established the Sham and CHF groups to better analyse differences between normal mice and CHF mice. Echocardiography manifested that the levels of EF, FS, LV+dP/dt_max_, LV−dP/dt_max_ were low in CHF mice, while the levels of LVIDd, LVIDs and LVEDP were high in CHF mice (Figure 1A–D). The outcome of ELISA implied that the contents of HF markers such as BNP, CTn‐I and Ang II were elevated; the SOD activity was impaired, while MDA content was increased; the levels of inflammatory factor TNF‐α, IL‐1β and IL‐6 were also elevated in CHF mice (Figure 1E–I).

The HE staining reflected the histopathological changes, which implied that the cardiomyocyte structure was normal without obviously broken fibers or inflammatory cells, and the nucleus was regular in normal mice; while the cardiomyocyte of CHF mice exhibited fiber breaking or thickening, accompanied by infiltration of inflammatory cells, and some of the nuclei showed deformity (Figure 1J). The results of Masson trichrome staining indicated that the fiber morphology of cardiomyocytes was regular and orderly in normal mice, while the CHF mice displayed disordered fiber and swollen cardiomyocytes, and the CVF was also obviously increased in CHF mice (Figure 1K). TUNEL staining unearthed more TUNEL‐positive cells in CHF mice, indicating the high apoptosis rate in CHF mice (Figure 1L). Furthermore, the apoptotic gene Caspase‐3 and Bax mRNA levels were augmented, while Bcl‐2 mRNA level was reduced in CHF mice (Figure 1M).

The above results manifested that the cardiac function was saliently impaired, and the inflammatory response was facilitated in CHF mice.

### 3.2 HOTAIR is depleted in CHF mice, and the cardiac function injury is improved after HOTAIR elevation while exacerbated after HOTAIR silencing

**FIGURE 2:**
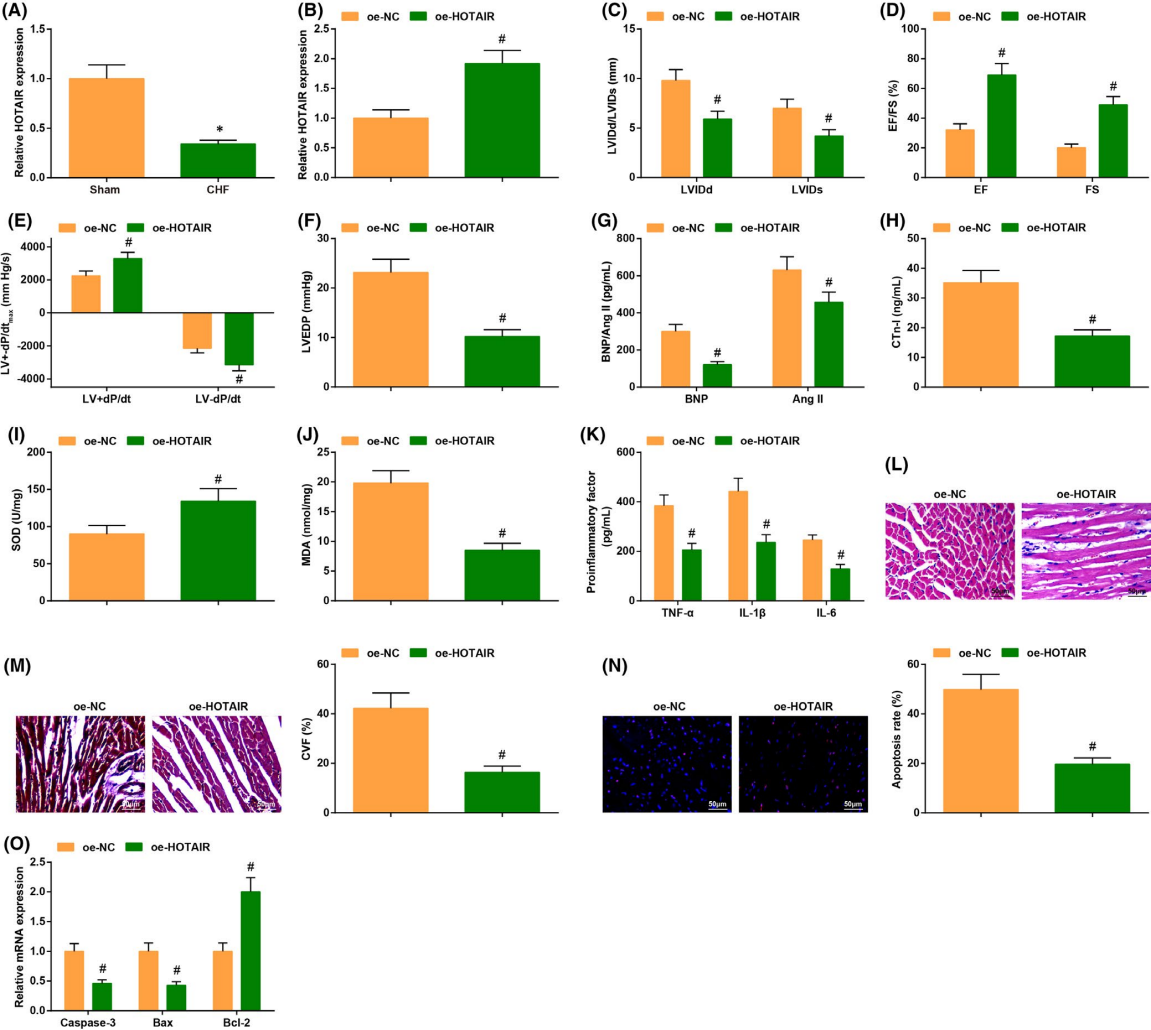
HOTAIR is depleted in CHF, and the cardiac injury is improved by HOTAIR elevation while exacerbated by HOTAIR silencing. (A/B) HOTAIR expression in CHF mice was detected by RT‐qPCR; C‐F, the cardiac function in CHF mice after injection of oe‐HOTAIR was detected by echocardiography; (G–K) the contents of cardiac function‐related factors and inflammatory factors as well as the oxidative stress response in CHF mice after injection of oe‐HOTAIR were examined by ELISA; (L) the cardiomyocyte damage in CHF mice after injection of oe‐HOTAIR was detected by HE staining; (M) cardiomyocyte fibrosis in CHF mice after injection of oe‐HOTAIR was detected by Masson trichrome staining; (N) the apoptosis rate in CHF mice after injection of oe‐HOTAIR was examined by TUNEL staining; (O) the levels of apoptosis‐related genes in CHF mice after injection of oe‐HOTAIR were assessed by RT‐qPCR. The data in the figure were all measurement data, and the value was expressed as mean ±standard deviation (Fig. A‐B, I‐J, L‐O: n = 6; other figures: n = 12); the unpaired t‐test was used for the comparison between two groups. * *p* < 0.05 vs. the Sham group; # *p* < 0.05 vs. the oe‐NC group

A previous study has validated that HOTAIR is modulated in non‐end‐stage HF patients, but the researches for probing the regulatory mechanism of HOTAIR in CHF were inadequate. To this end, the levels of HOTAIR in the myocardium of CHF mice were measured by RT‐qPCR. It was verified that HOTAIR was low‐expressed in the myocardium of CHF mice (Figure 2A).

We then established oe‐NC and oe‐HOTAIR adenovirus and injected them into the left ventricle of CHF mice. The changes of HOTAIR expression were detected by RT‐qPCR, reflecting augmented HOTAIR expression after injection of adenovirus solution with oe‐HOTAIR in CHF mice (Figure 2B).

In CHF mice injected with oe‐HOTAIR adenovirus, EF, FS, LV+dP/dt_max_, LV−dP/dt_max_ levels were increased; while the contents of LVIDd, LVIDs, LVEDP were depleted (Figure 2C–F); the levels of BNP, CTn‐I and Ang II were decreased; SOD activity was promoted while MDA content was reduced; the levels of TNF‐α, IL‐1β and IL‐6 were reduced compared with CHF mice injected with oe‐NC adenovirus (Figure 2G–K).

Through the histopathological observation and apoptosis detection, it was validated that the upregulation of HOTAIR in CHF mice could improve the abnormal fiber arrangement and mitigate inflammatory cell infiltration but decrease CVF and apoptosis rate induced by CHF (Figure 2L‐N), while caspase‐3 and Bax contents were decreased but Bcl‐2 level was enriched (Figure 2O).

These results evidenced that the overexpression of HOTAIR could saliently attenuate cardiac injury in CHF mice.

### 3.3 HOTAIR targets miR‐30a‐5p to restrain its expression

**FIGURE 3:**
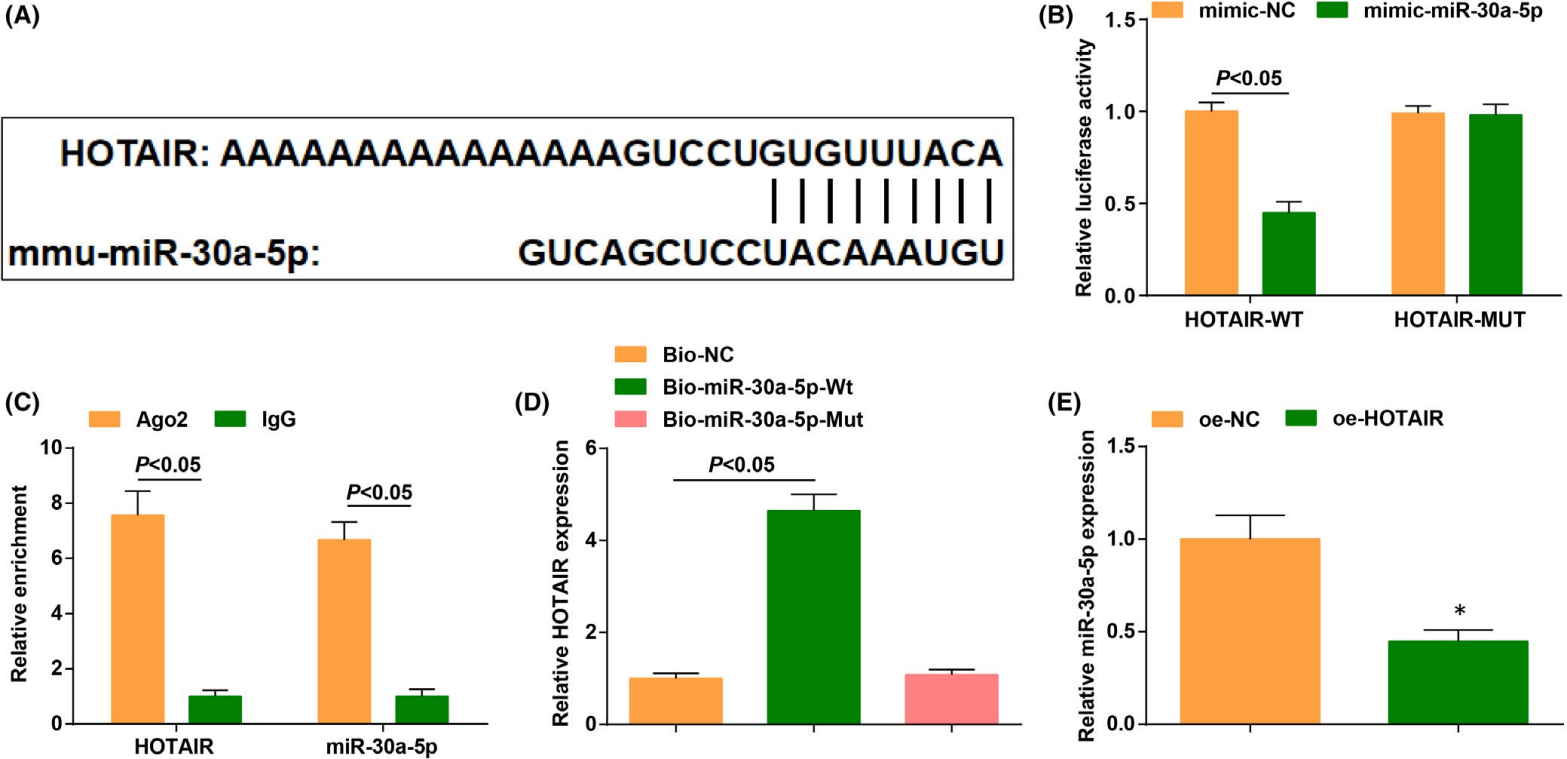
HOTAIR targets miR‐30a‐5p. (A) the binding sites of HOTAIR and miR‐30a‐5p were predicated by the bioinformatics website; (B) the targeting relationship between HOTAIR and miR‐30a‐5p was verified by dual luciferase reporter gene assay; (C) the relative enrichment of HOTAIR and miR‐30a‐5p was detected by RIP assay; (D) the combination pf HOTAIR and miR‐30a‐5p was validated by RNA pull‐down assay; (E) miR‐30a‐5p level in CHF mice after injection of oe‐HOTAIR was detected by RT‐qPCR. The data in the figure were all measurement data, and the value was expressed as mean ± standard deviation; the unpaired *t*‐test was used for the comparison between two groups; one‐way ANOVA was used for comparisons among multiple groups and Tukey's post hoc test was used for pairwise comparisons after one‐way ANOVA (Fig. D: *n* = 6; other figures: *N* = 3); * *p* < 0.05 vs. the oe‐NC group

The DIANA TOOLS bioinformatic website predicted that there were binding sites between HOTAIR and miR‐30a‐5p (Figure 3A).

To further verify the presence of binding sites, we conducted validation experiments. The result of dual luciferase reporter gene assay showed that the luciferase activity was repressed in cells co‐transfected with HOTAIR‐WT and mimic‐miR‐30a‐5p (Figure 3B). RIP assay implied that both HOTAIR and miR‐30a‐5p levels were increased in cells incubated with the RIP immunoprecipitation buffer containing magnetic beads conjugated with Ago2 (Figure 3C).

The results of RNA pull‐down assay disclosed that more HOTAIR co‐precipitated with Bio‐miR‐30a‐5p‐WT, indicating that miR‐30a‐5p bound to HOTAIR (Figure 3D).

Then, we performed RT‐qPCR on CHF mice injected with oe‐NC and oe‐HOTAIR adenovirus. The results uncovered that the overexpression of HOTAIR inhibited miR‐30a‐5p level (Figure 3E).

These findings above demonstrated that HOTAIR had a binding relation with miR‐30a‐5p.

### 3.4 MiR‐30a‐5p induction exacerbates cardiac injury in CHF mice

**FIGURE 4:**
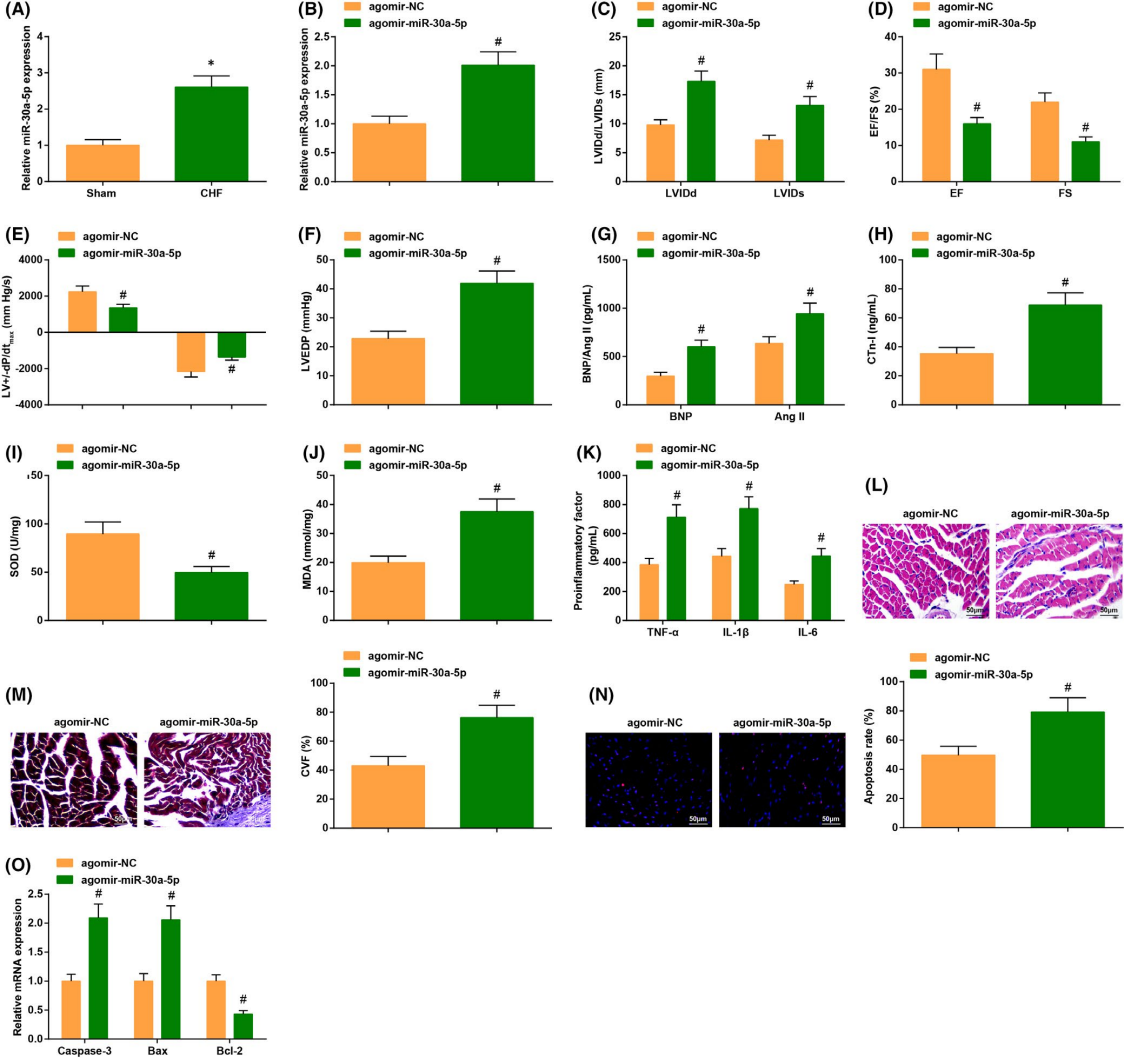
MiR‐30a‐5p induction exacerbates cardiac injury in CHF mice. (A/B) miR‐30a‐5p expression in CHF mice was detected by RT‐qPCR; (C–F) the cardiac function in CHF mice after injection with agomir‐miR‐30a‐5p was detected by echocardiography; (G–K) the contents of cardiac function‐related factors and inflammatory factors as well as the oxidative stress response in CHF mice after injection with agomir‐miR‐30a‐5p were examined by ELISA; (L) the cardiomyocyte damage in CHF mice after injection with agomir‐miR‐30a‐5p was detected by HE staining; (M) cardiomyocyte fibrosis in CHF mice after injection with agomir‐miR‐30a‐5p was detected by Masson trichrome staining; (N) the apoptosis rate in CHF mice after injection with agomir‐miR‐30a‐5p was examined by TUNEL staining; (O) the levels of apoptosis‐related genes in CHF mice after injection with agomir‐miR‐30a‐5p were assessed by RT‐qPCR. The data in the figure were all measurement data, and the value was expressed as mean ± standard deviation; the unpaired *t*‐test was used for the comparison between two groups (Fig. A‐B, I–J, L–O: *n* = 6; other figures: *n* = 12); * *p* < 0.05 vs. the Sham group; # *p* < 0.05 vs. the oe‐NC group

It has been found that miR‐30a‐5p is highly expressed in acute myocardial infarction. In light of this, we examined miR‐30a‐5p levels in the myocardium of mice, and it was verified that miR‐30a‐5p exhibited a high level in the myocardium of CHF mice (Figure 4A).

CHF mice were injected with the adenovirus vectors agomir‐NC and agomir‐miR‐30a‐5p. It was found that miR‐30a‐5p was elevated in CHF mice injected with agomir‐miR‐30a‐5p adenovirus (Figure 4B).

In CHF mice injected with agomir‐miR‐30a‐5p adenovirus vectors, EF%, FS, LV+dP/dt_max_ and LV‐dP/dt_max_ levels were reduced, while LVIDd, LVIDs and LVEDP levels were augmented (Figure 4C–F); the contents of BNP, CTn‐I and Ang II were amplified; SOD activity was decreased while MDA content was increased; the levels of TNF‐α, IL‐1β and IL‐6 were also elevated (Figure 4G–K).

Furthermore, it was also suggested that, in CHF mice injected with agomir‐miR‐30a‐5p adenovirus vectors, there were exacerbated abnormal fiber arrangement, inflammatory cell infiltration, elevated CVF and high apoptosis rate (Figure 4L–N); Caspase‐3 and Bax were enriched while Bcl‐2 was ablated (Figure 4O).

These discoveries verified that the elevation of miR‐30a‐5p aggravated the cardiac injury in CHF.

### 3.5 MiR‐30a‐5p can reverse the therapeutic effect of HOTAIR on cardiac injury in CHF mice

CHF mice were injected with oe‐NC +agomir‐NC, oe‐HOTAIR +agomir‐NC and oe‐HOTAIR +agomir‐miR‐30a‐5p, respectively.

**FIGURE 5:**
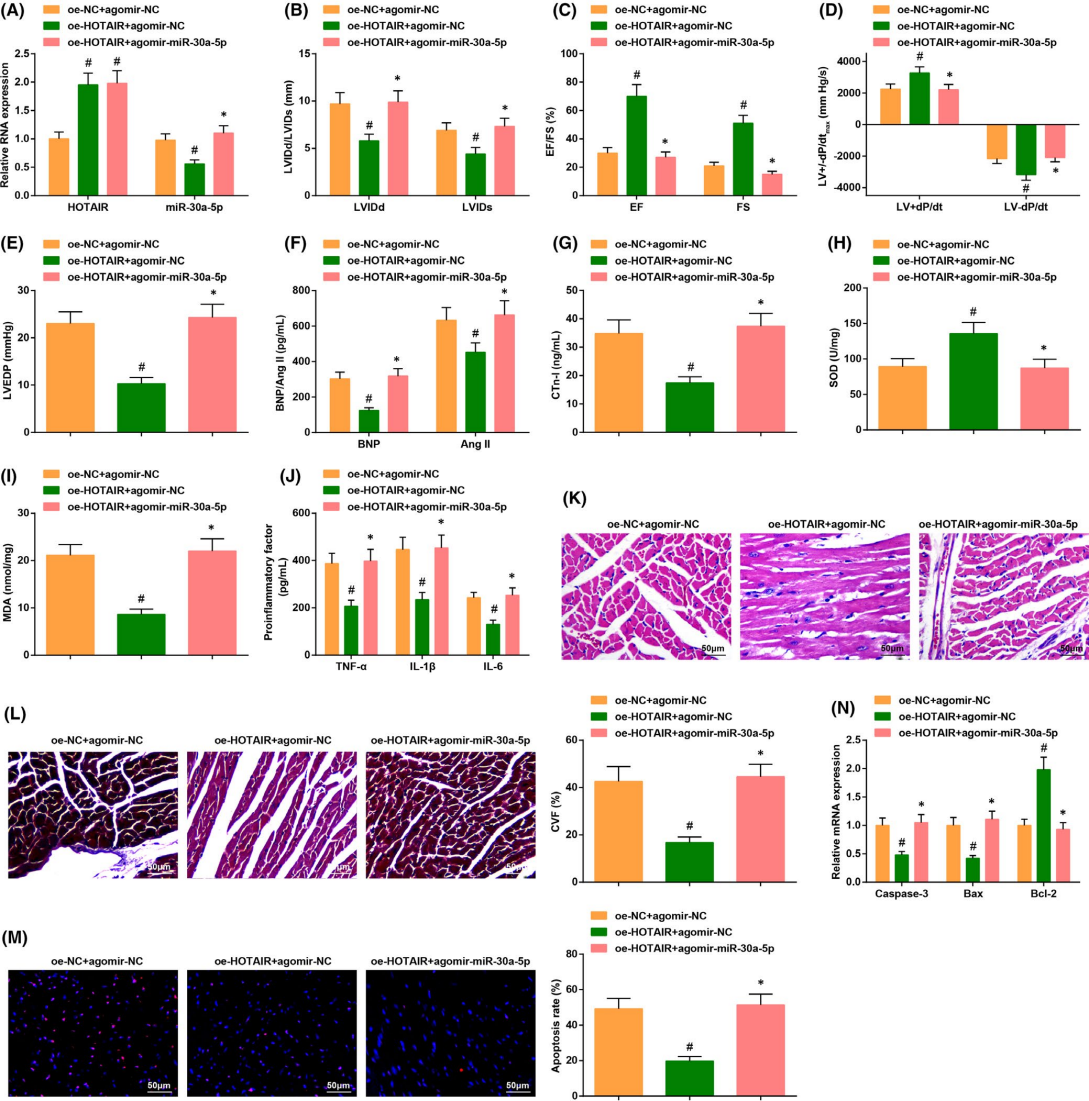
MiR‐30a‐5p can reverse the therapeutic effect of HOTAIR on cardiac injury in CHF mice. (A) KDM3A and miR‐30a‐5p levels in CHF mice after injection with oe‐HOTAIR +agomir‐miR‐30a‐5p were detected by RT‐qPCR; (B–E) the cardiac function in CHF mice after injection with oe‐HOTAIR +agomir‐miR‐30a‐5p was detected by echocardiography; (F–J) the contents of cardiac function‐related factors and inflammatory factors as well as the oxidative stress response in CHF mice after injection with oe‐HOTAIR +agomir‐miR‐30a‐5p were examined by ELISA; (K) the cardiomyocyte damage in CHF mice after injection with oe‐HOTAIR +agomir‐miR‐30a‐5p was detected by HE staining; (L) cardiomyocyte fibrosis in CHF mice after injection with oe‐HOTAIR +agomir‐miR‐30a‐5p was detected by Masson trichrome staining; (M) the apoptosis rate in CHF mice after injection with oe‐HOTAIR +agomir‐miR‐30a‐5p was examined by TUNEL staining; (N) the levels of apoptosis‐related genes in CHF mice after injection with oe‐HOTAIR +agomir‐miR‐30a‐5p were assessed by RT‐qPCR. The data in the figure were all measurement data, and the value was expressed as mean ± standard deviation; one‐way ANOVA was used for comparisons among multiple groups and Tukey's post hoc test was used for pairwise comparisons after one‐way ANOVA (Figure A, H–I, K–M: *n* = 6; other figures: *n* = 12); * *p* < 0.05 vs. the oe‐HOTAIR + agomir‐NC group; # *p* < 0.05 vs. the oe‐NC +agomir‐NC group

Through the same series of experiments, we found that the upregulation of miR‐30a‐5p could reverse the therapeutic effect of HOTAIR in CHF mice, leading to the inhibited recovery of cardiac function, irregular fiber arrangement, increased CVF level and accelerated cardiomyocyte apoptosis rate (Figure 5A‐N).

These results implied that miR‐30a‐5p overexpression reversed the effects of elevated HOTAIR on improving cardiac injury.

### 3.6 MiR‐30a‐5p targets KDM3A

**FIGURE 6:**
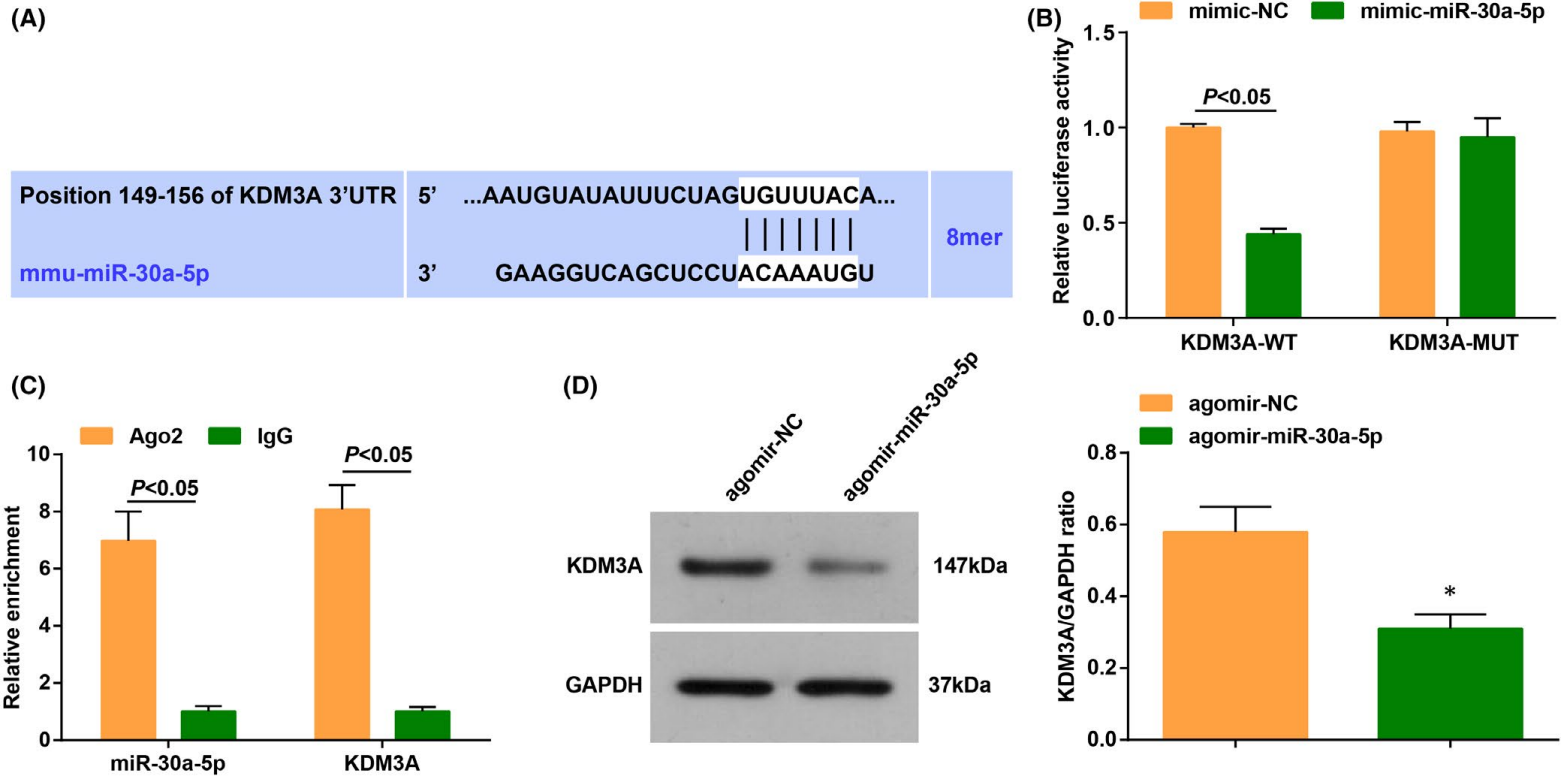
MiR‐30a‐5p targets KDM3A. (A) The binding sites between miR‐30a‐5p and KDM3A were predicated by bioinformatic website TargetScan; (B) the targeting relationship between miR‐30a‐5p and KDM3A was verified by dual luciferase reporter gene assay; (C) the enrichment of miR‐30a‐5p and KDM3A was examined by RIP assay; (D) KDM3A expression in CHF mice after injection with agomir‐miR‐30a‐5p was detected by Western blot assay. The data in the figure were all measurement data, and the value was expressed as mean ± standard deviation; the unpaired *t*‐test was used for the comparison between two groups (Fig. D: *n* = 6; other figures: *N* = 3); * *p* < 0.05 vs. the agomir‐NC group

To probe whether there existed a targeting relation between miR‐30a‐5p and KDM3A, it was predicated that miR‐30a‐5p has a targeting relation with KDM3A through the bioinformatics website TargetScan website (Figure 6A).

Similarly, two experiments were performed to further verify the binding relation. The dual luciferase reporter gene assay showed that the induction of mimic‐miR‐30a‐5p inhibited the luciferase activity of KDM3A‐WT (Figure 6B). RIP assay revealed that miR‐30a‐5p and KDM3A expression augmented drastically in cells incubated with the RIP immunoprecipitation buffer containing magnetic beads conjugated with Ago2 (Figure 6C).

The results of Western blot assay also implied that miR‐30a‐5p elevation suppressed KDM3A expression in mice injected with agomir‐miR‐30a‐5p (Figure 6D).

The results above clarified that miR‐30a‐5p had a targeting relation with KDM3A.

### 3.7 Downregulation of BNIP3 can reverse the therapeutic effect of KDM3A on cardiac function in CHF mice

Previous researches have validated that KDM3A can open the gene interior, bind to BNIP3 and regulate its expression, thus reducing cell apoptosis. Thus, we hypothesized that the regulatory effects of KDM3A and BNIP3 might enlighten CHF treatment.

**FIGURE 7:**
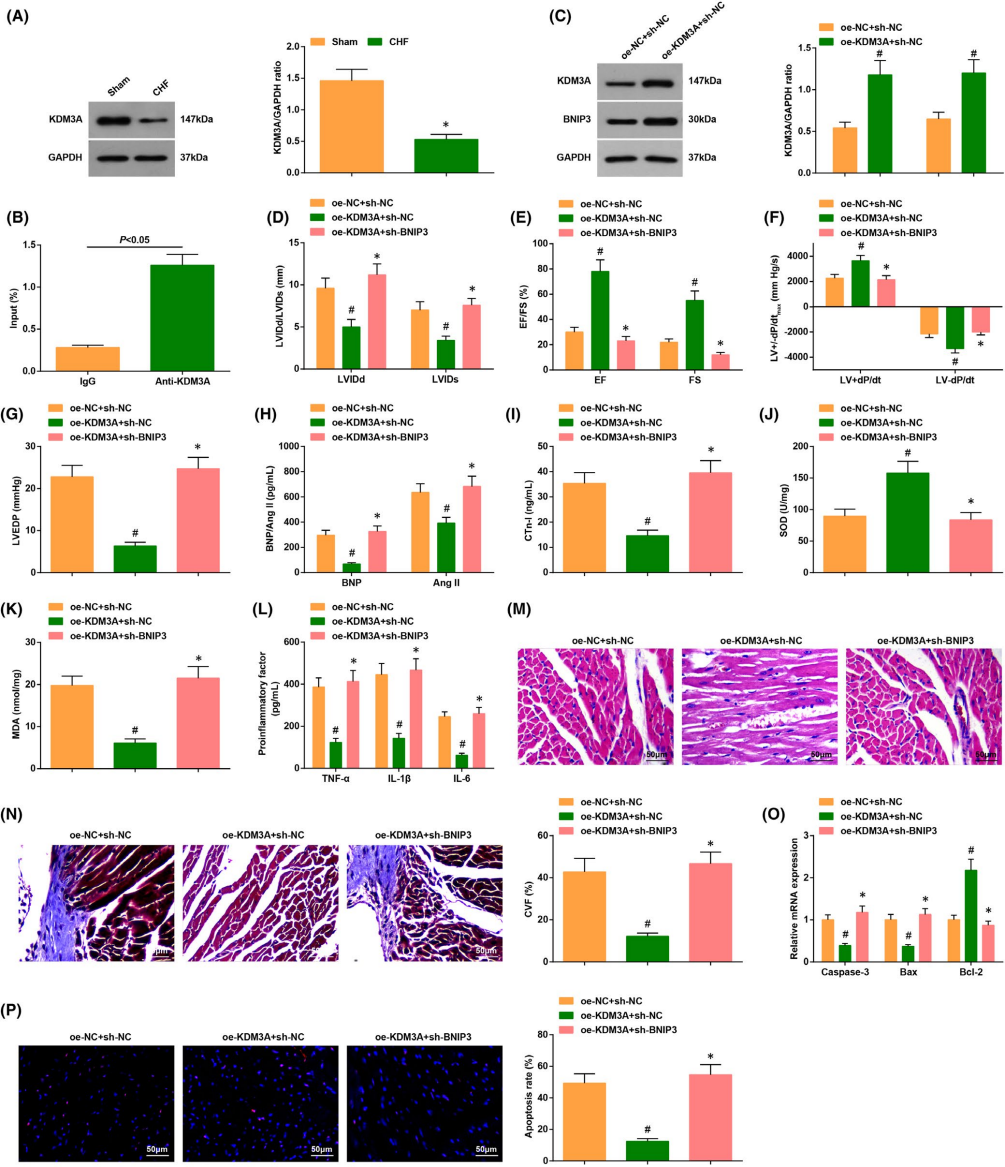
Downregulation of BNIP3 can reverse the therapeutic effect of KDM3A on cardiac injury in CHF mice. (A) KDM3A protein levels in CHF mice were detected; (B) the binding of KDM3A to BNIP3 was assessed by ChIP assay; (C) KDM3A and BNIP3 protein levels in CHF mice were detected; (D‐G) the cardiac function in CHF mice after being treated with oe‐KDM3A + sh‐BNIP3 was detected by echocardiography; (H‐K) the contents of cardiac function‐related factors and inflammatory factors as well as the oxidative stress response in CHF mice after being treated with oe‐KDM3A + sh‐BNIP3 were examined by ELISA; (l) the cardiomyocyte damage in CHF mice after being treated with oe‐KDM3A + sh‐BNIP3 was detected by HE staining; (M) cardiomyocyte fibrosis in CHF mice after being treated with oe‐KDM3A + sh‐BNIP3 was detected by Masson trichrome staining; (N) the apoptosis rate in CHF mice after being treated with oe‐KDM3A + sh‐BNIP3 was examined by TUNEL staining; (O) the levels of apoptosis‐related genes in CHF mice after being treated with oe‐KDM3A + sh‐BNIP3 were assessed by RT‐qPCR. The data in the figure were all measurement data, and the value was expressed as mean ± standard deviation; the unpaired t‐test was used for the comparison between two groups; one‐way ANOVA was used for comparisons among multiple groups and Tukey's post hoc test was used for pairwise comparisons after one‐way ANOVA (A‐C, I‐J, L‐O: *n* = 6; other figures: *n* = 12); # *p* < 0.05 vs. the oe‐NC + sh‐NC group; * *p* < 0.05 vs the oe‐KDM3A + sh‐NC group

First, we explored levels of KDM3A in CHF mice. The results of Western blot assay showed that KDM3A expression was reduced in CHF mice (Figure 7A,B).

The outcome of ChIP assay implied that KDM3A could bind to the promoter of BNIP3, indicating that KDM3A could regulate BNIP3 expression (Figure 7C).

CHF mice were treated with adenovirus solution that contained oe‐NC + sh‐NC, oe‐KDM3A + sh‐NC and oe‐KDM3A + sh‐BNIP3. It came out that KDM3A and BNP3 levels were elevated after injection with oe‐KDM3A + sh‐NC by Western blot assay (Figure 7D,E).

In CHF mice injected with oe‐KDM3A + sh‐NC, the CHF was improved as reflected by the mitigated oxidative stress, inflammatory factor levels, pathological change and cardiomyocyte apoptosis, while the induction of BNIP3 reversed the therapeutic effects of elevated KDM3A on CHF through the result of a series of experiments (Figure 7F–R).

These outcomes uncovered that the downregulation of BNIP3 reversed the therapeutic effects of augmented KDM3A on cardiac injury of CHF mice.

## 4 DISCUSSION

**FIGURE 8:**
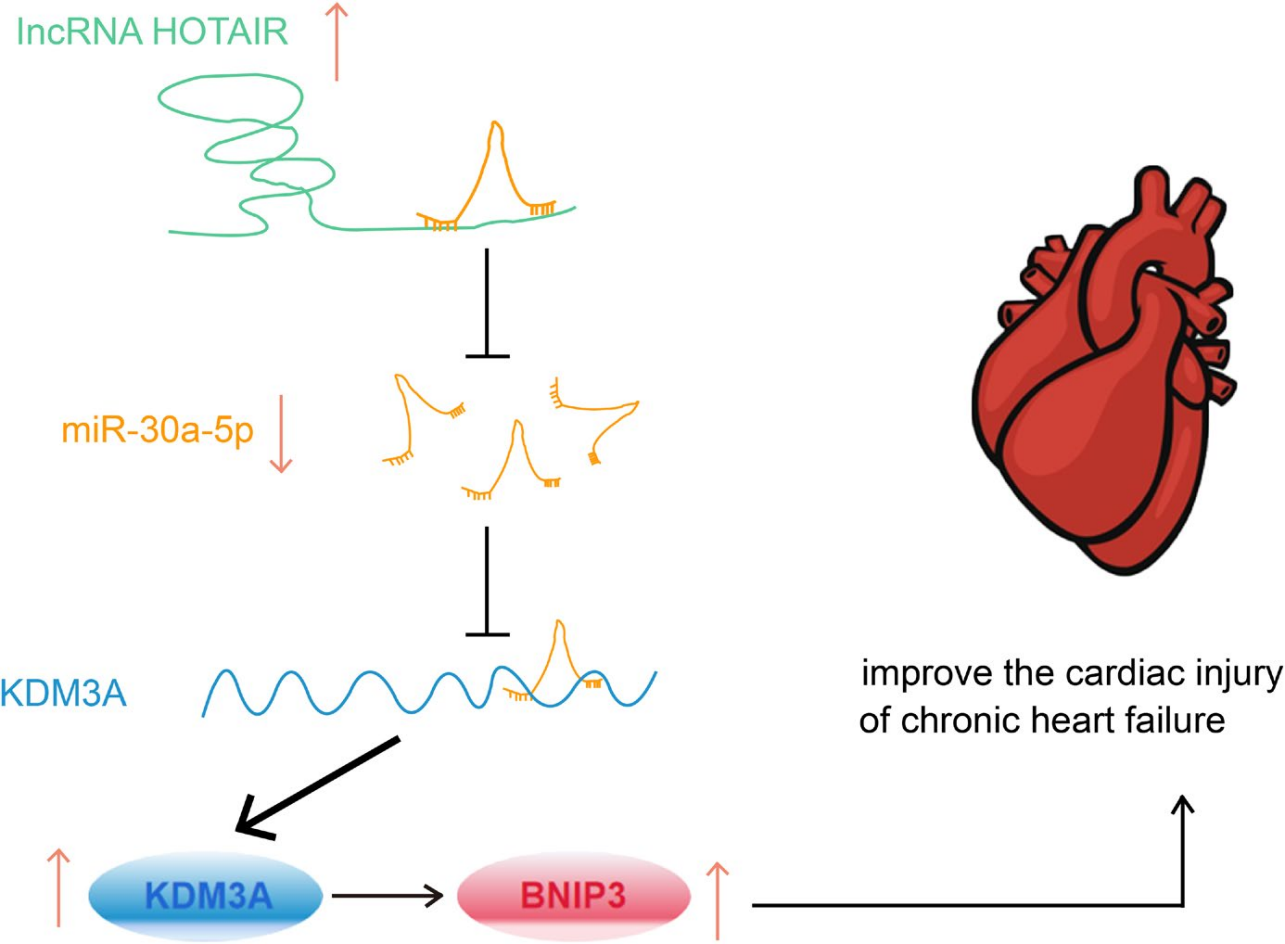
Upregulation of HOTAIR improves cardiac injury in CHF via modulating miR‐30a‐5p to target KDM3A. In CHF mice with cardiac injury, HOTAIR and KDM3A levels were ablated while miR‐30a‐5p was elevated. miR‐30a‐5p targets KDM3A, and KDM3A binds to BNIP3. Up‐regulated HOTAIR improves cardiac dysfunction in CHF mice, resulting in decreased apoptosis of cardiomyocytes and improved cardiac dysfunction

HF is a chronic and debilitating disease that jeopardizes the ability of the heart to meet the demands for cardiac output.^2^ This study focused on the regulatory mechanism of HOTAIR on cardiac function injury in CHF mice. Collectively, it was demonstrated that HOTAIR could attenuate the cardiac function injury in CHF mice via targeting miR‐30a‐5p to regulate KDM3A (Figure 8).

This study initially demonstrated that HOTAIR was depleted in the myocardium of CHF mice, and the upregulation of HOTAIR could ameliorate the cardiac function injury, oxidative stress, pathological change, inflammatory responses and cardiomyocyte apoptosis in CHF mice. In line with our findings, Lai et al. have also elucidated that HOTAIR is low expressed in heart tissue and cardiomyocytes of mice with cardiac hypertrophy, while the elevation of HOTAIR suppresses the level of hypertrophic markers, thus blocking the progression of cardiac hypertrophy. Similarly, HOTAIR augmentation can also attenuate oxidative stress and H_2_O_2_‐induced cell apoptosis as well as facilitate the viability of H9c2 cells. Furthermore, Gao et al. have verified the low expression of HOTAIR in the serum of patients with an acute myocardial infraction, and the hypoxia‐induced cardiomyocyte apoptosis can be dramatically decelerated through the elevation of HOTAIR. Such finding has been further evidenced by Zhang et al., who have elucidated that the amplified HOTAIR can mitigate myocardial infraction or hypoxia‐induced cardiomyocytes apoptosis. In addition, in atrial fibrillation, HOTAIR also exhibits a low level in cardiomyocytes, and the amplification of HOTAIR can effectively restrain the development of atrial fibrillation.

Moreover, it was confirmed that HOTAIR targeted miR‐30a‐5p. Then, our study further manifested that miR‐30a‐5p displayed a high level in the cardiomyocytes and myocardium of CHF mice. The elevation of miR‐30a‐5p exacerbated the cardiac function injury, oxidative stress, inflammation as well as accelerated cell apoptosis in CHF mice. Some corresponding studies also have verified the related regulatory mechanism of miR‐30a‐5p in CHF or heart‐related diseases. For instance, Ding et al. have clarified that miR‐30a‐5p shows an upward trend with the incidence of HF, suggesting miR‐30a‐5p functions as a novel biomarker for the diagnosis of CHF and related diseases. Furthermore, miR‐30a‐5p has been validated to display a high level in patients with developed left ventricular dysfunction and HF symptoms after acute myocardial infarction, and miR‐30a‐5p also displays a negative monotonic correlation with left ventricular ejection fraction value. Similarly, Marques et al. have elucidated that the failing hearts tend to absorb miR‐30a‐5p, which further modulate the pathophysiology of cardiac failure as well as pathways related to disease progression, such as fibrosis.

Furthermore, miR‐30a‐5p was predicted to have a binding relation with KDM3A. In this study, it was demonstrated that KDM3A was ablated in the myocardium of CHF mice; the augmented KDM3A contributed to mitigating the cardiac function injury, oxidative stress, pathological change, inflammatory responses and the cardiomyocyte apoptosis in CHF mice. Several preceding studies also concluded the function of KDM3A in heart related and other diseases. For instance, Liu et al. have revealed that KDM3A is critical for modulating macrophage polarization, phagocytosis and migration to participate in the cardiac repair process and left ventricular remodelling post. Concretely, another study about myocardial infarction also has illustrated that KDM3A displays a low level, and KDM3A elevation can alleviate myocardial fibrosis, reduce inflammatory cytokine contents as well as decrease apoptosis cells. In addition, this research further discovered that it was also validated that KDM3A could bind to the promoter of BNIP3 through the ChIP assay as well as modulate BNIP3 level. In consistent with this finding, Zhang et al. have reported that KDM3A can modulate BNIP3 expression by promoting histone demethylation in the BNIP3 promoter region.

Collectively, this study verifies that HOTAIR and KDM3A are decreased while miR‐30a‐5p is enriched in CHF mice, and HOTAIR effectively ameliorates the cardiac function injury in CHF mice via regulating miR‐30a‐5p to target KDM3A. The current discovery makes a contribution for exploring novel therapeutic strategies for CHF by highlighting the importance of the HOTAIR/miR‐30a‐5p/KDM3A axis. However, the size of study samples and grouping should be increased to get more precise data for a convincing conclusion.

## CONFLICT OF INTEREST

The authors have no relevant financial or non‐financial interests to disclose.

## AUTHOR CONTRIBUTIONS

**Xiaoyun Zhang:** Conceptualization (equal); writing – original draft (equal). **Yakun Gao:** Conceptualization (equal); data curation (equal). **Hongyu Wu:** Formal analysis (equal); investigation (equal). **Yong Mao:** Resources (equal); Software (equal); supervision (equal). **Yanqing Qi:** Writing – review and editing (equal).

## Supporting information

Table S1

## Data Availability

The data that support the findings of this study are available from the corresponding author upon reasonable request.

## References

[1] Tanai E , Frantz S . Pathophysiology of heart failure. Compr Physiol. 2015;6(1):187‐214. 26756631 10.1002/cphy.c140055

[2] Rogers C , Bush N . Heart failure: pathophysiology, diagnosis, medical treatment guidelines, and nursing management. Nurs Clin North Am. 2015;50(4):787‐799. 26596665 10.1016/j.cnur.2015.07.012

[3] Baman JR , Ahmad FS . Heart failure. JAMA. 2020;324(10):1015. 32749448 10.1001/jama.2020.13310

[4] Tamargo J , Caballero R , Delpon E . New drugs in preclinical and early stage clinical development in the treatment of heart failure. Expert Opin Investig Drugs. 2019;28(1):51‐71. 10.1080/13543784.2019.1551357 30523722

[5] Huang Y . The novel regulatory role of lncRNA‐miRNA‐mRNA axis in cardiovascular diseases. J Cell Mol Med. 2018;22(12):5768‐5775. 30188595 10.1111/jcmm.13866 PMC6237607

[6] Bhan A , Mandal SS . LncRNA HOTAIR: A master regulator of chromatin dynamics and cancer. Biochim Biophys Acta. 2015;1856(1):151‐164. 26208723 10.1016/j.bbcan.2015.07.001 PMC4544839

[7] Liu L , et al. Myocardin‐related transcription factor A (MRTF‐A) regulates integrin beta 2 transcription to promote macrophage infiltration and cardiac hypertrophy in mice. Cardiovasc Res. 2021. Online ahead of print. 10.1093/cvr/cvab110 33752236

[8] Ye S , et al. LCZ696 attenuated doxorubicin‐induced chronic cardiomyopathy through the TLR2‐MyD88 complex formation. Front Cell Dev Biol. 2021;9:654051. 33928085 10.3389/fcell.2021.654051 PMC8076895

[9] Cao Y , et al. Astragalus polysaccharide suppresses doxorubicin‐induced cardiotoxicity by regulating the PI3k/Akt and p38MAPK pathways. Oxid Med Cell Longev. 2014;2014:674219. 25386226 10.1155/2014/674219 PMC4216718

[10] Liu J , et al. Extracellular vesicles‐encapsulated let‐7i shed from bone mesenchymal stem cells suppress lung cancer via KDM3A/DCLK1/FXYD3 axis. J Cell Mol Med. 2021;25(4):1911‐1926. 33350586 10.1111/jcmm.15866 PMC7882949

[11] Che M , et al. Long noncoding RNA HCG18 inhibits the differentiation of human bone marrow‐derived mesenchymal stem cells in osteoporosis by targeting miR‐30a‐5p/NOTCH1 axis. Mol Med. 2020;26(1):106. 33176682 10.1186/s10020-020-00219-6 PMC7656763

[12] Lin QY , et al. LncRNA PVT1 acts as a tumor promoter in thyroid cancer and promotes tumor progression by mediating miR‐423‐5p‐PAK3. Cancer Manag Res. 2020;12:13403‐13413. 33408513 10.2147/CMAR.S283443 PMC7779291

[13] Zhang Y , Li Y . Long non‐coding RNA NORAD contributes to the proliferation, invasion and EMT progression of prostate cancer via the miR‐30a‐5p/RAB11A/WNT/beta‐catenin pathway. Cancer Cell Int. 2020;20(1):571. 33292272 10.1186/s12935-020-01665-2 PMC7694907

