# Supplementary material for: LncRNA HOX transcript antisense RNA mitigates cardiac function injury in chronic heart failure via regulating microRNA‐30a‐5p to target KDM3A
Source: J Cell Mol Med. 2022 Jan 26;26(5):1473–85. doi: 10.1111/jcmm.17160 (PMC8899154; doi:10.1111/jcmm.17160)
Supplement: Supplementary file 1 — Table S1 [file JCMM-26-1473-s001.docx]

**Supplementary Table 1** Primer sequences used for q-PCR

| Genes | Primer sequences (5’-3’) |
| --- | --- |
| HOTAIR | F: GGCTGCCTGAGTTCTTTTGC |
|  | R: TGCGGTGGAGATAGATGTGC |
| miR-30a-5p | F: TGTAAACATCCTCGACTGGAAG |
|  | R: Universal primer |
| Caspase-3 | F: GGAGTCTGACTGGAAAGCCGAA |
|  | R: CTTCTGGCAAGCCATCTCCTCA |
| Bax | F: AGGATGCGTCCACCAAGAAGCT |
|  | R: TCCGTGTCCACGTCAGCAATCA |
| Bcl-2 | F: CCTGTGGATGACTGAGTACCTG |
|  | R: AGCCAGGAGAAATCAAACAGAGG |
| U6 | F: CTCGCTTCGGCAGCACA |
|  | R: AACGCTTCACGAATTTGCGT |
| GAPDH | F: AAGCCCATCACCATCTTCCAGGAG |
|  | R: AGCCCTTCCACAATGCCAAAG |

Notes: F, forward; R, reverse; HOTAIR, long noncoding RNA HOX transcript antisense RNA; miR-30a-5p, microRNA-30a-5p; Bax, B cell lymphoma-2-associated X; Bcl-2, B cell lymphoma-2; GAPDH, glyceraldehyde-3-phosphate dehydrogenase.
